# Strengths-Based Leadership and Turnover Intention: The Roles of Felt Obligation for Constructive Change and Job Control

**DOI:** 10.3389/fpsyg.2022.786551

**Published:** 2022-04-13

**Authors:** Xixi Chu, He Ding, Lihua Zhang, Zhuyi Angelina Li

**Affiliations:** ^1^School of Management and Economics, Beijing Institute of Technology, Beijing, China; ^2^School of Economics and Management, North China Electric Power University, Beijing, China; ^3^School of Labor and Human Resources, Renmin University of China, Beijing, China; ^4^School of Business, Renmin University of China, Beijing, China

**Keywords:** strengths-based leadership, felt obligation for constructive change, job control, turnover intention, substitutes for leadership theory

## Abstract

This study draws on the substitutes for leadership theory to investigate the association of strengths-based leadership with employee turnover intention and the mediating role of felt obligation for constructive change and the moderating role of job control in the linkage. Data were collected using a three-wave survey from a sample of 317 employees working in a variety of enterprises in China. The multiple regression analyses with bootstrapping procedure were utilized to examine the proposed hypotheses. The results indicate that strengths-based leadership negatively relates to turnover intention and felt obligation for constructive change partially mediates the relationship between strengths-based leadership and turnover intention. Furthermore, job control, acting as a substitute for strengths-based leadership, negatively moderates the indirect relationship between strengths-based leadership and turnover intention *via* felt obligation for constructive change. This study contributes to the literature of strengths-based leadership and the substitutes for leadership theory by enhancing our understanding of the effect of job control.

## Introduction

According to a report released by 51JOBS, China’s largest human resource service provider, the overall Chinese employee turnover rate in 2021 was 18.8% and the voluntary turnover rate reached 14.1%, which are much higher than other countries. Frequent staff turnover entails costs to organizations, increases loss of tacit knowledge and social capital, and can trigger other employees’ emotional instability and turnover contagion within the organization (Heavey et al., 2013; Itzchakov et al., 2022). Hence, it is important to clarify the factors influencing Chinese employee turnover and reduce their turnover intention. Turnover intention, defined as the possibility that an employee will leave the employing institution and seek other job opportunities (Mobley, 1977), is the strongest single predictor of actual turnover (Hom et al., 2012). A recent meta-analysis argued that leaders are particularly valuable to employee retention (Rubenstein et al., 2018). Prior studies have shown that many leadership styles, such as ethical leadership (Demirtas and Akdogan, 2015) and transformational leadership (Oh and Chhinzer, 2021), can effectively reduce employee turnover intention. However, there remains a dearth of literature on the relationship between strengths-based leadership and employee turnover intention.

Strengths-based leadership represents a positive leadership style that leaders seek to promote the identification, development, and deployment of strengths in their own and their followers in order to improve individual and organizational performance (Ding and Yu, 2021b). Extant study has demonstrated that strengths-based leadership encourages employees to use their own strengths at work (Ding and Yu, 2021a), so as to improve their task performance (Ding et al., 2020) and innovative behaviors (Ding and Yu, 2020a) and contribute to employee psychological well-being (Ding and Yu, 2021b). These findings provide promising evidence for the relationship between strengths-based leadership and employee turnover intention. Indeed, a 2-year case study of St Lucie Medical Center in Florida indicated that properly leveraging top leaders’ and employees’ strengths can significantly reduce attrition rate and increase employee engagement and job satisfaction (Burkus, 2011). In addition, a study based on 7 industries in 45 countries showed that strengths interventions can reduce turnover by 26- to 72-point in high-turnover organizations and by 6- to 16-point in low-turnover organizations (Rigoni and Asplund, 2016). Unfortunately, few empirical studies have examined whether strengths-based leadership can affect employee turnover intention and the potential mechanisms accounting for this relationship are underdeveloped. Therefore, we aim to redress these gaps by proposing a moderated mediation model regarding strengths-based leadership and turnover intention.

Substitutes for leadership theory are a theoretical framework developed on the basis of path-goal theory to explain the contingency relationship between leadership behaviors and outcome variables, which are distinguished from other leadership contingency theories by highlighting the importance of situational factors (Kerr and Jermier, 1978). Based on the substitutes for leadership theory, individual characteristics affected by leader behavior (e.g., subordinate professional orientation) can transmit the influence or importance of the leader behavior on some performance or consequence (Dionne et al., 2002). Felt obligation for constructive change is an individual proactive psychological state, which reflects a willingness to put more effort into the work, as well as bring about improvement and new procedures and correct broader problems (Fuller and Hester, 2010). It can be considered as such an individual characteristic (Fuller and Hester, 2010). Specifically, when employees perceive that their leaders give them more autonomy to deploy their strengths at work, their need for autonomy can be fulfilled (Kong and Ho, 2016). The increased job autonomy can lead individuals to believe that their work product is a function of their own decisions and efforts, thus enhancing their feeling of obligation for their work product (Hackman and Oldham, 1980; Ding and Yu, 2021b) and ultimately forming greater intrinsic work motivation, greater job satisfaction, and greater concern about the quality of their work (Fuller and Hester, 2010). Hence, it is feasible to expect that strengths-based leadership has a negative association with employee turnover intention *via* felt obligation for constructive change.

Additionally, study has also pointed that the effectiveness of leadership depends on work characteristics (Wang and Cheng, 2010; Ding and Yu, 2020a). Job control as an important work characteristic is defined as the extent to which a job gives employees substantial freedom, independence, and discretion in scheduling and performing their job (Hackman, 1976). According to Yperen and Hagedoorn (2003), enhancing job control cannot only reduce work stress, but also increase employee intrinsic work motivation. As such, we expect job control to act as a moderator of the relationship between strengths-based leadership and felt obligation for constructive change. Substitutes for leadership theory argue that certain organizational characteristics (i.e., characteristics of the organization, the subordinate, and the task) can substitute or neutralize the effects of the leader’s behaviors (Kerr and Jermier, 1978). Higher levels of job control positively affect employee felt obligation for constructive change in that job control provides freedom, independence, and discretion to employees on their day-to-day job, leading to a greater satisfaction with the need for autonomy and job experience (Frank and David, 2001), which helps shape their feeling of obligation (Hackman and Oldham, 1980). Therefore, we expect that job control will act as a substitute for strengths-based leadership, such that the direct relationship of strengths-based leadership with felt obligation for constructive change and the indirect relationship of strengths-based leadership with turnover intention *via* felt obligation for constructive change will be weaker under higher, rather than lower, levels of job control.

Taken together, this study offers three contributions to previous literature on the strengths-based leadership and turnover intention. First, by presenting felt obligation for constructive change as a mediator of strengths-based leadership, we extend the works by Ding and Yu (2020a) and Ding and Quan (2021) on how individual characteristics transmit the influence or importance of strengths-based leadership behaviors on some performance or consequence, providing a new insight into the psychological mechanism underlying the relationship between strengths-based leadership and turnover intention. In doing so, we address the call from Dionne et al. (2002) to advance the substitutes for leadership theory by examining indirect leader effects that may be mediated by substitutes. Second, in contrast to other leadership theories, substitutes for leadership theory recognize the role of followers in the leadership process (Mostafa, 2018). By assessing whether employees’ job control may substitute the role of strengths-based leadership in the relationships among strengths-based leadership, felt obligation for constructive change, and turnover intention, this study attempts to expand the literature on substitutes for leadership theory, highlights the potential role of job control as an important boundary condition of strengths-based leadership, and helps to find a way through which organization can enhance the effectiveness of strengths-based leadership in terms of increased felt obligation for constructive change and reduced turnover intention. Third, by extending the substitutes for leadership theory to the field of strengths-based leadership and substantiating its relevance, we address the concern of overreliance of prior strengths-based leadership research on the conservation of resources, self-determination, and job demands-resources theories.

## Theory and Hypotheses

### Strengths-Based Leadership and Turnover Intention

Strengths-based leadership, as an innovative and positive leadership style, brings about greater gains of efficiency, productivity, and organizational success by continuously building the strengths of leaders and their followers (Burkus, 2011). According to Rath and Conchie (2008), strengths-based leadership has three basic tenants: (1) invest their time and energy in their followers’ strengths; (2) build well-rounded teams to meet the requirements for strengths in executing, influencing, relationship building, and strategic thinking; and (3) understand followers’ need to build trust, hope, and optimism. More importantly, strengths-based leaders do not ignore their own and followers’ weaknesses, but rather focus on building their own and team members’ strengths and minimizing the negative effects of weaknesses (Burkus, 2011; Van Woerkom et al., 2016).

Prior studies have demonstrated that strengths-based leadership has a conducive effect on employee work engagement (Burkus, 2011) and psychological well-being (Ding and Yu, 2020a). However, little is known about the relationship between strengths-based leadership and employee turnover intention. This study posits that strengths-based leadership negatively relates to employee turnover intention. On one hand, individuals who have opportunities to regularly leverage their strengths at work are more likely to have higher life satisfaction and are more engaged in work (Winseman, 2002). More impressively, a study of St Lucie Medical Center in Florida noted that building teams that properly use employees’ strengths can significantly reduce employee attrition rate and dramatically increase the satisfaction of both the physicians and patients (Burkus, 2011). Thus, strengths-based leadership focusing on the identification, development, and deployment of strengths in leaders and followers may negatively relate to employee turnover intention. On the other hand, strengths-based leadership behaviors, such as aligning employees’ strengths with work tasks and devoting more time and energy to their strengths (Rath and Conchie, 2008), can create a positive climate to improve employees’ task performance (Ding et al., 2020) and innovative behaviors (Ding and Yu, 2020a) and even psychological well-being (Ding and Yu, 2021b), which have a negative effect on employee turnover intention (Oi et al., 2015). Therefore, based on the above reasoning, the following hypothesis was derived:

Hypothesis 1: Strengths-based leadership negatively relates to turnover intention.

### Felt Obligation for Constructive Change as a Mediator

Felt obligation for constructive change, a malleable psychological state, has been defined as “an individual’s belief that he or she is personally obligated to bring about constructive change” (Morrison and Phelps, 1999, p. 407). A substantial body of studies have found that felt obligation for constructive change cannot only effectively stimulate both the promotive (Carnevale et al., 2019) and prohibitive voices (Jian et al., 2012), but also improve proactive role performance, such as change-oriented organizational citizenship behavior (Lopez-Dominguez et al., 2013) and innovation (Parker and Collins, 2010). Importantly, employees with a strong sense of obligation for constructive change can also experience higher levels of personal accomplishment and satisfaction (Morrison and Phelps, 1999) because when employees feel obligation for constructive change at work, they will experience greater intrinsic work motivation and are more likely to engage in work as “responsible citizens” (Jian et al., 2012). These positive outcomes induced by felt obligation for constructive change are negatively correlated with turnover intention (Mobley, 1977).

Given the importance of felt obligation for constructive change to organizations and employees, many researchers have attempted to identify the antecedents of felt obligation for constructive change. For example, Lopez-Dominguez et al. (2013) found that resource availability can effectively enhance employees’ felt obligation for constructive change. This study postulates that strengths-based leadership contributes to increased employee felt obligation for constructive change. First, Fuller and Hester (2010) pointed out that employees who possess more work resources are more likely to feel personal obligation for constructive change. In this sense, strengths-based leadership as a crucial work resource (Ding and Yu, 2021a) might positively influence employees’ felt obligation for constructive change. Second, strengths-based leaders provide employees more autonomy to use strengths at work, which satisfy employees’ need for autonomy (Ding and Yu, 2021a). Autonomy as a core job characteristic can foster feelings of obligation for constructive change (Hackman and Oldham, 1980; Fuller and Hester, 2010). Third, employees will experience higher leader–member exchange relationship when leaders help them to identify, develop, and leverage their strengths at work (Ding and Yu, 2020b). Employees in high-quality leader–member exchange relationship will feel responsible for initiating constructive change in the organization (Carnevale et al., 2019). Therefore, it is possible to expect that strengths-based leadership is positively related to employees’ felt obligation for constructive change. Furthermore, considering that the substitutes for leadership theory suggest that individual characteristics can transmit the influence or importance of the leader behavior on some performance or consequence (Dionne et al., 2002), it is reasonable to assume that strengths-based leadership contributes to employee felt obligation for constructive change and in turn to reduced employee turnover intention. Taken together, the following hypothesis is offered:

Hypothesis 2: Felt obligation for constructive change mediates the relationship between strengths-based leadership and turnover intention.

### Job Control as a Moderator

Job control, sometimes called decision latitude (Doef and Maes, 1999), refers to the influence of employees on their actions and work conditions (Frese, 1989). Employees with higher job control are apt to experience higher creative self-efficacy (Du et al., 2018), positive mental health (Crown, 2007), and lower levels of workload and burnout (Leiter and Maslach, 2004). Job control is an important work contextual factor (Holman et al., 2002; Oi et al., 2015). Previous studies focused not only on the consequences of job control, but also on the moderating role of job control (Doef and Maes, 1999). For instance, Holman et al. (2002) demonstrated that job control moderates the association of perceived intensity with well-being.

According to the substitutes for leadership theory, certain individual, task, and organizational characteristics, acting as “substitutes for leadership,” can impact the influence of the leaders’ behaviors (Kerr and Jermier, 1978). Extant study has found that when job control is introduced as a moderator, the significant relationship between transformational leadership and followers’ innovative behaviors will become insignificant (Lopez-Dominguez et al., 2013). As such, we argue that a higher level of job control, which is viewed as an important work characteristic (Hackman, 1976), may act as a substitute for strengths-based leadership.

Howell et al. (1986) proposed three criteria for acting as a substitute: (1) the leadership and substitute variables must be related to the outcome variable; (2) the substitute must have a significant positive impact on the outcome variable; and (3) at different levels of the substitute (i.e., higher or lower), the relationship between the leadership and the outcome variable must be weakened. In alignment with these three standards, first, strengths-based leadership (Ding and Yu, 2021a) and job control (Crown, 2007), respectively, meet employees’ needs for autonomy, which is a key antecedent of felt obligation for constructive change (Fuller and Hester, 2010), thus felt obligation for constructive change may be positively predicted by strengths-based leadership and job control. Second, Fuller and Hester (2010) indicated that employees who have greater control over their jobs are more likely to have feelings of obligation for constructive change, which provides promising evidence for the positive relationship between job control and felt obligation for constructive change. Third, we posit that the positive relationship between strengths-based leadership and felt obligation for constructive change will be weaker under higher levels of job control as job control substitutes the strengths-based leadership. In a state of high job control, employees have the substantial freedom, independence, and discretion in scheduling and performing their work (Hackman and Oldham, 1980), such as autonomously introducing their own strengths to the work. Thus, when strengths-based leadership is lower, the autonomy provided by higher levels of job control can still provide opportunities for employees to work on their strengths (Kong and Ho, 2016), thereby fostering employees’ felt obligation for constructive change (Parker et al., 1997; Parker, 2003). On the contrary, in the absence of control over work, employees rely more on their leaders to provide autonomy to utilize their strengths at work and employees’ need for autonomy can be satisfied (Ding and Yu, 2020a), thus driving felt obligation for constructive change. Taken together, we postulate that since job control acts as a substitute for strengths-based leadership, the relationship between strengths-based leadership and felt obligation for constructive change should be weaker under the condition of higher levels of job control.

Hypothesis 3: Job control acts as a substitute for strengths-based leadership, such that the magnitude of the positive relationship between strengths-based leadership and felt obligation for constructive change will be weaker under higher, rather than lower, levels of job control.

The above propositions involve an integrative framework in which employee felt obligation for constructive change mediates the relationship of strengths-based leadership with employee turnover intention and the association of strengths-based leadership with employee felt obligation for constructive change is contingent on job control. Accordingly, we further expect that job control as a substitute of strengths-based leadership negatively moderates the mediational effect of felt obligation for constructive change on the relationship between strengths-based leadership and employee turnover intention. Hence, the following hypothesis is offered:

Hypothesis 4: Job control acts as a substitute for strengths-based leadership, such that the magnitude of the indirect relationship between strengths-based leadership and employee turnover intention through felt obligation for constructive change will be weaker under higher, rather than lower, levels of job control.

**FIGURE 1:**
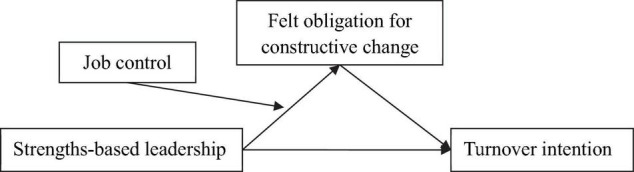
The proposed conceptual model.

The proposed conceptual model was depicted in Figure 1.

## Materials and Methods

### Sample and Procedure

Participants in this study were Chinese employees working in diverse organizations. Our inclusion criteria were as follows: (a) participants should work as a full-time in their organizations and (b) participants should take part in this survey voluntarily. The first author of this study contacted 75 alumni working in a variety of enterprises (e.g., financial industry) in China to freely participate in this study and requested them to invite their colleagues to join this study. With the help of these alumni, we recruited 584 employees who met our requirements and volunteered to take part in the survey. We gathered study data at three points in time and paid 5 renminbi (RMB) as a reward for each questionnaire. In the process of data collection, we promised that information related to participants would be treated as confidential strictly. Cell phone numbers were used to match data from three phases.

We used a Chinese social network mobile application called Wechat to distribute online questionnaires. To minimize common method variance (CMV), we used a three-wave longitudinal data. At time 1, 584 participants completed questionnaire regarding demographic characteristics, strengths-based leadership scale, and cell phone numbers. We chose to collect the second wave data after 1 month according to a study by Podsakoff et al. (2003). At time 2, we sent the link of the questionnaire concerning felt obligation for constructive change, job control scales, and cell phone numbers and received 425 questionnaires, demonstrating 72.77% response rate relative to T1. According to a study by Podsakoff et al. (2003), the time intervals between measurements for job attitudes are at least 2–3 months and we chose to collect the third wave data after 3 months. At time 3, we invited participants who responded in the second phase to complete a questionnaire about turnover intention scale and cell phone numbers. Eventually, 317 valid matched data were obtained, indicating 54.28% response rate relative to T1 and 74.59% response rate relative to T2. Among them, 44.50% were male, 93.70% had Bachelor’s degree or above, 84.5% had worked in current organization for more than 7 years, and 80.04% were 30–50 years old. Table 1 shows the sample distribution.

**TABLE 1:** Sample distribution (*N* = 317).

Variables	Categories	Frequency (%)	Variables	Categories	Frequency (%)
Education	Specialist or under	6.3	Tenure (years)	1–3	5.7
	Bachelor	55.5		4–6	9.8
	Master	34.1		7–9	10.4
	Doctor	4.1		10–13	30.9
Age	Less than 30	14.2		More than 13	43.2
	30–40	62.8	Gender	Male	44.5
	41–50	17.7		Female	55.5
	51–60	3.8			
	More than 60	1.6			

### Measures

The original strengths-based leadership scale, felt obligation for constructive change scale, and job control scales were in English. We translated these scales into Chinese following the translation and back translation procedures suggested by Brislin (1970). All the items of scales were rated on a 5-point Likert scale (*1* = *strongly disagree* to *5* = *strongly agree*).

#### Strengths-Based Leadership

We used 10 items of strength-based leadership scale developed by Ding and Yu (2021b). One sample item was “My leader is good at using my strengths.” The Cronbach’s α was 0.918.

#### Felt Obligation for Constructive Change

We measured felt obligation for constructive change with 5 items from a study by Jian et al. (2012). One sample item was “I feel a personal obligation to produce constructive suggestions to help the organization achieve its goals.” The Cronbach’s α was 0.919.

#### Job Control

Job control was measured with 11-item scale developed by Wall et al. (1996), including timing control items and method control items. One sample item was “Do you decide on the order in which you do things.” The Cronbach’s α was 0.895.

#### Turnover Intention

Turnover intention was assessed by the 4-item scale developed by Weng (2010). This scale referred to a study by Mobley et al. (1978). One sample item was “I will probably leave this company in a year.” The Cronbach’s α was 0.833.

#### Control Variables

Prior studies have shown that age, education, and tenure were correlated with turnover intention (Tschopp et al., 2014). With respect to this study, age (*r* = −0.25, *p* < 0.01), educational level (*r* = 0.11, *p* < 0.05), and tenure (*r* = −0.26, *p* < 0.01) were significantly related to turnover intention. According to the suggestion of Tschopp et al. (2014), although studies focusing on turnover intentions did not show a significant effect between gender and turnover intention, we decided to control for gender as well. As such, age, gender, and educational and tenure levels were considered as control variables in this study.

## Results

### Confirmatory Factor Analysis

We conducted confirmatory factor analysis (CFA) to examine the discriminant validity between strengths-based leadership, felt obligation for constructive change, job control, and turnover intention before testing our predictions. We chose fit indexes of χ^2^/df (should be less than 3), standardized root mean square residual (SRMR) (should be less than 0.08), comparative fit index (CFI) (should be more than 0.09), Tucker–Lewis index (TLI) (should be more than 0.09), and root mean square error of approximation (RMSEA) (should be less than 0.08) to evaluate the fit of the model, as recommended by previous studies (Hu and Bentler, 1999; Byrne, 2013). As shown in Table 2, the results of CFA showed that the four-factor measurement model exhibited the best fit to the data (χ^2^ = 307.35, df = 113, χ^2^/df = 2.72, SRMR = 0.07, CFI = 0.95, TLI = 0.94, and RMSEA = 0.04). In sum, the four-factor measurement model had a better fit to the data than alternative models.

**TABLE 2:** Results of confirmatory factor analyses (CFAs): comparison of measurement models (*N* = 317).

Models	χ^2^	df	χ^2^/df	RMSEA	CFI	TLI	SRMR
Four-factor model	307.35	113	2.72	0.07	0.95	0.94	0.04
Three-factor model^a^	937.51	116	8.08	0.15	0.78	0.74	0.11
Two-factor model^b^	1630.57	118	13.82	0.20	0.59	0.52	0.12
One-factor model^c^	2043.58	119	17.17	0.23	0.47	0.40	0.15

*^a^Strengths-based leadership and felt obligation for constructive change combined into one factor.*

*^b^Strengths-based leadership, felt obligation for constructive change and job control combined into one factor.*

*^c^All combined into one factor.*

Although this study collected data at three time points, self-report questionnaire may bring about CMV. This study utilized Harman’s single factor test and CFA to test the degree of common method bias (Podsakoff et al., 2003). Harman’s single factor test showed that 33.58% of the variance could be explained by the first principal factor, which was less than 40%. Besides, CFA revealed that the χ^2^ was significantly improved (Δχ^2^ = 1736.23, *p* < 0.05) compared to the four-factor measurement model to the one-factor measurement model. Therefore, the CMV of this study was not serious.

In addition, Cheung and Rensvold (2002) pointed that χ^2^ is easily influenced by the sample size and is overly sensitive to the sample when the sample size is greater than 200. They recommend comparing CFI to choose the model. The sample size of this study was 317, which was greater than 200. Therefore, it was necessary to test the degree of common method bias by comparing CFI. According to a study by Podsakoff et al. (2003), we constructed an unmeasured method factor and loaded the method factor on all the indices of strengths-based leadership, job control, felt obligation for constructive change, and turnover intention. Analytical results showed that CFI index of this five-factor measurement model (χ^2^ = 227.66, df = 97, χ^2^/df = 2.35, SRMR = 0.07, CFI = 0.96, TLI = 0.95, and RMSEA = 0.04) has no significant change, exhibiting no better fit to the data than the four-factor measurement model. Accordingly, the CMV was not a big concern for influencing the accuracy of our results.

### Descriptive Statistics

Table 3 reports the mean, SD, and correlations for study variables. In Table 3, the results indicated that the study variables (i.e., strengths-based leadership, job control, felt obligation for constructive change, and turnover intention) were all significantly related with each other. These results provided preliminary evidence for our hypotheses.

**TABLE 3:** Descriptive statistics and correlations (*N* = 317).

	Variables	Mean	SD	1	2	3	4	5	6	7
1	Age	2.16	0.77	–						
2	Gender	1.56	0.50	0.03	–					
3	Education	2.36	66	−0.32**	0.04	–				
4	Tenure	3.96	1.20	0.64**	–0.11	−0.23**	–			
5	Strengths-based leadership	3.56	0.79	–0.07	–0.05	0.11	–0.01	–		
6	Job control	3.49	0.61	–0.05	–0.06	0.06	0.05	0.39**	–	
7	Felt obligation for constructive change	3.82	0.71	–0.01	–0.07	0.08	0.10	0.43**	0.50**	–
8	Turnover intention	2.78	0.91	−0.25**	–0.09	0.11*	−0.26**	−0.30**	−0.22**	−0.28**

*p < 0.05, **p < 0.01.

### Hypotheses Testing

To examine our hypotheses, the multiple regression analyses was carried out in SPSS version 22.0, which was combined with bootstrapping analyses with bias-corrected CI based on 5,000 bootstrap samples. Results are shown in Table 4.

**TABLE 4:** Results of process analysis (*N* = 317).

Variables	Felt obligation for constructive change		Turnover intention		
	Model 1	Model 2	Model 3	Model 4	Model 5
Age	–0.02	0.02	−0.14*	–0.13	−0.14*
Gender	–0.03	–0.03	−0.13*	−0.13*	−0.13**
Education	0.06	0.04	0.07	0.06	0.08
Tenure	0.13	0.08	−0.18**	−0.15*	−0.15*
SBL	0.42***	0.28***	−0.32***		−0.25***
FOCC				−0.27***	−0.17**
JC		0.35***			
SBL × JC		−0.13**			
*R* ^2^	0.20	0.33	0.20	0.17	0.22
*F*	15.29***	21.76***	15.06***	12.44***	14.35***

*p < 0.05, **p < 0.01, ***p < 0.001.

*SBL, strengths-based leadership; FOCC, felt obligation for constructive change; JC, job control; SBL × JC, interaction of strengths-based leadership and job control.*

Hypothesis 1 postulated that strengths-based leadership was negatively related to employee turnover intention. As shown in model 3 in Table 4, the relationship between strengths-based leadership and turnover intention was significant (β = −0.32, *p* < 0.001), indicating that strengths-based leadership negatively relates to employee turnover intention. Hypothesis 1 is supported.

Hypothesis 2 supposed that felt obligation for constructive change mediated the relationship between strengths-based leadership and turnover intention. As shown in model 5 in Table 4, coefficient of felt obligation for constructive change was significant (β = −0.17, *p* < 0.01). PROCESS (model 4) was applied to further examine the indirect effect. Results showed that the indirect effect of strengths-based leadership on turnover intention through felt obligation for constructive change was significant [indirect effect = −0.08, CI: (−0.14, −0.03)]. Additionally, the direct effect between strengths-based leadership and turnover intention was also significant [direct effect = −0.23, CI: (−0.35, −0.12)]. Accordingly, we could conclude that felt obligation for constructive change partially mediated the relationship of strengths-based leadership with turnover intention.

**FIGURE 2:**
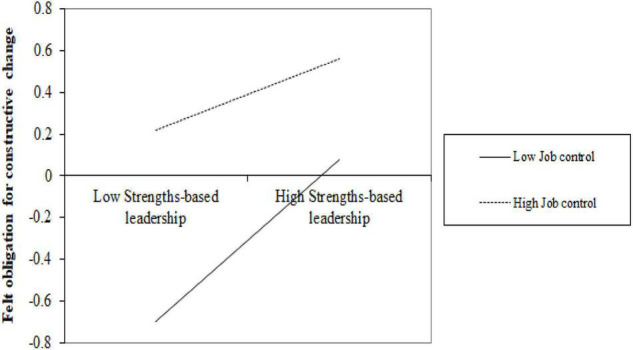
Moderating effect of self-efficacy on the relationship between strengths use and job crafting.

Hypothesis 3 expected that job control could weaken the positive relationship between strengths-based leadership and felt obligation for constructive change. PROCESS (model 1) was used to test this hypothesis. Strengths-based leadership and job control were standardized before conducting the analysis. Model 2 in Table 4 indicated that strengths-based leadership positively related to felt obligation for constructive change (β = 0.28, *p* < 0.001), job control positively related to felt obligation for constructive change (β = 0.35, *p* < 0.001), and the interaction term of strengths-based leadership and job control was negatively related to felt obligation for constructive change (β = −0.13, *p* < 0.01). The data analysis showed that job control satisfies the three criteria for acting as a substitute of strengths-based leadership, which supported Hypothesis 3. As in Figure 2, we conducted the interaction slope analyses to show the relationship of strengths-based leadership and felt obligation for constructive change when the level of job control was low (M − SD) and high (M + SD) to further elaborate on this interaction effect. Specifically, the positive relationship between strengths-based leadership and felt obligation for constructive change was weaker for employees with a higher level of job control [β = 0.17, CI: (0.05, 0.23)] than for employees with a lower level of job control [β = 0.38, CI: (0.25, 0.51)], which further supported Hypothesis 3.

Hypothesis 4 assumed that job control could weaken the mediating effect of felt obligation for constructive change on the relationship between strengths-based leadership and turnover intention. PROCESS (model 7) was utilized to test the moderated mediation effect. The results showed that the moderated mediation effect was significant [β = 0.02, CI: (0.002, 0.04)], supporting hypothesis 4. Furthermore, we conducted the difference test of the indirect effects. The results showed that the mediation effect of felt obligation for constructive change was significantly different at high (M + SD) and low (M − SD) level of job control [difference estimate = 0.04, CI: (0.003, 0.08)]. Specifically, the mediating effect of felt obligation for constructive change was weaker for employees with a higher level of job control [β = −0.02, CI: (−0.07, −0.004)] than for employees with a lower level of job control [β = −0.06, CI: (−0.12, −0.02)], which further supported hypothesis 4.

## Discussion

This study conducts a survey of 317 employees working in various organizations and investigates the relationship between strengths-based leadership and employee turnover intention and the mediating role of employee felt obligation for constructive change as well as the moderating role of job control in this relationship. As predicted, all the hypotheses are supported by the study data. This study offers several theoretical contributions and practical implications.

### Theoretical Contributions

This study offers three contributions to previous literature on the strengths-based leadership and employee turnover intention theories and study. First, this study is the first study to empirically examine the linkage between strengths-based leadership and employee turnover intention. The results demonstrate that strengths-based leadership is negatively related to employee turnover intention. This finding is consistent with previous study suggesting that strengths-based leadership has a negative relationship with employee turnover (Burkus, 2011). The negative relationship of strengths-based leadership with employee turnover intention can be elaborated by the fact that strengths-based leaders can build a positive climate contributing to employees’ task performance (Ding et al., 2020), innovative behavior (Ding and Yu, 2020a), and even psychological well-being (Ding and Yu, 2021b), thereby increasing employees’ work engagement and reducing their turnover intention (Burkus, 2011). Therefore, this study offers a new piece of empirical evidence for the relationship between strengths-based leadership and employee turnover intention.

Second, by investigating the potential mediating role of employee felt obligation for constructive change in the relationship between strengths-based leadership and employee turnover intention, this study contributes to a better understanding of why strengths-based leadership is related to employee turnover intention. Importantly, this study also addresses the call from a study by Dionne et al. (2002) to advance the substitutes for leadership theory by examining indirect leader effects that may be mediated by substitutes such as individual characteristic. Our findings indicate that employee felt obligation for constructive change acting as a substitute of strengths-based leadership partially mediates the relationship between strengths-based leadership and employee turnover intention. This phenomenon is aligned to the theoretical notion that strengths-based leaders help subordinates to identify, develop, and leverage their strengths at work, which can build better relationship between supervisors and subordinates. To reciprocate supervisors, subordinates make more effort to fulfill job responsibilities and achieve a wide range of positive outcomes (Ding and Yu, 2020b), such as lower turnover intention. Given that, this study helps to better understand the potential psychological mechanism underlying the linkage of strengths-based leadership and employee turnover intention.

Third, one of the key criticisms of strengths-based leadership study has been the lack of boundary conditions in explaining its effects (Ding and Yu, 2020b). In response to this call, this study has advanced our understanding of strengths-based leadership through the substitutes for leadership theory by providing insights into job control as a substitute of strengths-based leadership and highlighting the importance of understanding the boundary conditions of the strengths-based leadership. Therefore, similar to previous studies that servant leadership (Eva et al., 2021) and transformational leadership (Walter and Bruch, 2010) are not equally applicable to all the situations, we argue that although strengths-based leadership is beneficial for both the employees and organizations, this may not be the case for any organizational context. On the contrary, there may be cases in which employee turnover intention is reduced, but this is not related to the direct contribution of strengths-based leadership. In fact, our findings indirectly validate the study of Zappalà and Toscano (2019) that the significant correlation between transformational leadership and innovative behaviors is not more significant when job control is introduced as a moderator. In summary, drawing on the substitutes for leadership theory, this study extends prior study on strengths-based leadership by demonstrating the importance of job control on the effects of leaders’ behaviors.

### Practical Implications

This study has three overarching recommendations for organizations. First, regardless of job control, leaders who exhibit strengths-based leadership behaviors would generally elicit higher levels of felt obligation for constructive change and lower levels of turnover intention among their employees. For organizations, it is necessary to foster, accelerate, and reinforce strengths-based leadership behaviors and provide strengths-based leadership development programs for their managers. For leaders, there is a need to improve the ability to identify, develop, and utilize their won and employees’ strengths in the workplace.

Second, based on the finding about the mediational effect of felt obligation for constructive change on the relationship between strengths-based leadership and employee turnover intention, organizations can reduce employees’ turnover intention by facilitating employees’ felt obligation for constructive change. The specific strategies of nurturing employees’ felt obligation for constructive change can follow the suggestions proposed by Fuller and Hester (2010), namely, job autonomy, position in organization hierarchy, accessing to resource, and accessing to information.

Third, since job control is found to be a substitute for strengths-based leadership in the direct relationship of strengths-based leadership and employee felt obligation for constructive change and the indirect relationship of strengths-based leadership and employee turnover intention through felt obligation for constructive change, it is recommended that organizations provide employees with freedom, independence, and discretion in their daily work to positively impact job control. Allowing employees freedom, independence, and discretion in organizing work and procedures help to protect the organization from lower levels of felt obligation for constructive change and higher levels of employee turnover intention, if strengths-based leadership behaviors are not embedded within the organization.

### Limitations and Future Study Directions

This study is not without limitations. First, we collected study data from a single source. However, single-resource data were suitable for this study because the outcome variable was employee’s work attitude (i.e., turnover intention), which cannot be measured by other resources, except self-report (Mobley et al., 1978). In order to address the CMV concerns related to self-report data, we have taken multiple remedies and the statistical tests showed that CMV was unlikely to be a problem. For future studies, it is recommended that longitudinal study design or experimental study should be conducted to capture the fluctuation of leaders’ approach over a period of time commensurate with the changing context.

Second, this study only analyzed strengths-based leadership approach without controlling other competing leadership approaches. Antonakis (2017, p. 10) argued that failing “to control for these competing constructs will engender omitted variable bias and does not inform us of the incremental validity of the construct.” Prior studies have shown the positive relationships of servant leadership (Liden et al., 2014), ethical leadership (Elci et al., 2012), and transformational leadership (Green et al., 2013) with employee turnover intention. However, these leadership styles were not considered as control variables in this study. Thus, future studies need to examine the incremental predictive validity of strengths-based leadership in terms of employee turnover intention after controlling for these competing leadership approaches.

Third, this study focused on employees working in various organizations in China, which might limit the generalizability of our findings. Hence, future study needs to investigate the proposed theoretical model in a cross-organizational and cultural background. In addition, this study has only started to reveal how the work characteristics impact the influence of strengths-based leadership. Future study could broaden it to include organizational context, organizational strategy, to name just a few. Furthermore, we expect that job control would moderate the linkage of strengths-based leadership with other employee outcomes such as job satisfaction, trust, and thriving at work. A greater understanding of how the work characteristics might act as substitutes for strengths-based leadership and its subsequent effects on the individual, team, and organizational outcomes will make strengths-based leadership more credible.

## Data Availability Statement

The original contributions presented in the study are included in the article/supplementary material, further inquiries can be directed to the corresponding author.

## Author Contributions

XC collected the research data, designed the research, and wrote the manuscript. HD analyzed and interpreted the data, and together with LZ and ZL amended the manuscript. All authors contributed to the article and approved the submitted version.

## Conflict of Interest

The authors declare that the research was conducted in the absence of any commercial or financial relationships that could be construed as a potential conflict of interest.

## Publisher’s Note

All claims expressed in this article are solely those of the authors and do not necessarily represent those of their affiliated organizations, or those of the publisher, the editors and the reviewers. Any product that may be evaluated in this article, or claim that may be made by its manufacturer, is not guaranteed or endorsed by the publisher.
